# OncoVee™-MiniPDX-Guided Anticancer Treatment for Gastric Cancer Patients With Synchronous Liver Metastases: A Retrospective Cohort Analysis

**DOI:** 10.3389/fonc.2021.757383

**Published:** 2022-01-03

**Authors:** Yutong Ge, Xin Zhang, Wei Liang, Cuiju Tang, Dongying Gu, Junfeng Shi, Xiaowei Wei

**Affiliations:** ^1^ Department of Oncology, Nanjing First Hospital, Nanjing Medical University, Nanjing, China; ^2^ Department of Gastrointestinal Surgery, Qilu Hospital of Shandong University, Jinan, China

**Keywords:** MiniPDX, gastric cancer, hepatic metastases, survival, response, OncoVee

## Abstract

**Background:**

It is estimated that 35% of gastric cancer patients appear with synchronous distant metastases—the vast majority of patients presenting with metastatic hepatic disease. How to choose the most appropriate drugs or regimens is crucial to improve the prognosis of patients. We conducted this retrospective cohort analysis to evaluate the efficacy of OncoVee™-MiniPDX-guided treatment for these patients.

**Methods:**

Gastric cancer patients with liver metastases (GCLM) were enrolled. Patients were divided into MiniPDX and control group according to their wishes. In the observation group, the OncoVee™-MiniPDX model was conducted to screen the most sensitive drug or regimens to determine the clinical administration. Meanwhile, patients were treated with regular medications in the control group according to the guidelines without the MiniPDX model. The primary endpoint was overall survival (OS), and the secondary outcomes included objective response rate (ORR), disease control rate (DCR), and progression-free survival (PFS).

**Results:**

A total of 68 patients with GCLM were included, with the observation and control groups of 21 and 47 patients, respectively. The baseline characteristics of patients were balanced between these two groups. MiniPDX drug sensitivity tests were associated with the increased use of targeted drugs when compared with the control group (33.3 *vs.* 0%, p=0.032). Median OS was estimated to be 9.4 (95% CI, 7.9–11.2) months and 7.9 (95% CI, 7.2–8.7) months in the observation and control group, respectively. Both univariate (control group *vs.* MiniPDX group: HR=2.586, 95% CI= 1.362–4.908, p=0.004) and multivariate regression analyses (Control group *vs.* MiniPDX group: adjusted HR (aHR)=4.288, 95% CI= 1.452–12.671, p=0.008) showed the superiority of the observation group on OS. Similarly, MiniPDX-based regiments significantly improve the PFS of these cases (median PFS 6.7 months *vs.* 4.2 months, aHR=2.773, 95% CI=1.532–3.983, p=0.029). ORR and DCR were also improved in MiniPDX group comparing with control group (ORR, 57.14 *vs.* 25.53%, p=0.029; DCR: 85.71 *vs.* 68.08%, p=0.035).

**Conclusion:**

OncoVee™-MiniPDX model, which was used to select drugs to guide antitumor treatment, was promising to prolong survival and improve the response rate of patients with GCLM. Further well-designed studies are needed to confirm the clinical benefits of MiniPDX.

## 1 Introduction

Gastric cancer (GC) is one of the most common malignant tumors and the fifth leading cause of cancer-related death worldwide (1). The situation is even grimmer in China, which accounts for about half of the morbidity and mortality associated with stomach disease (1, 2). Although the age-adjusted incidence and mortality rates in gastric cancer have decreased during the last decades, the relative survival has only witnessed a modest increase compared to improvements in many other gastrointestinal cancers (3). Metastatic spread is fatal to patients by leading to mass-effects and failures of physiological homeostasis. During the last two decades, the proportion of gastric cancer patients with synchronous metastases has increased to over 35–40% (4), with the vast majority of patients presenting with metastatic hepatic disease.

Hepatic resection should always be considered as an option for gastric cancer patients with liver metastases. However, some patients with GC are not suitable for hepatic resection, for whom adjuvant chemotherapy or molecular targeted therapy would be a choice. Newly developed cytotoxic agents represented by S-1 show promising activity for patients with metastases (5). How to choose the most sensitive antitumor drugs is crucial to improve the prognosis of patients.

Cancer research relies on interrogation model systems that reflect the biology of human tumors. Primary cell culture from human tumors has been a traditional approach to cancer research, but significant differences between *in vitro* cell culture environments and *in vivo* tumor environments have raised concerns that these cell lines may not be fully representative of human tumors (6). Patient-derived xenograft (PDX) model, injecting the tumor fragments from the patient into immunodeficient mice directly, has become a powerful method for preclinical drug evaluation (7–9). The advantage of PDX models to cell lines or genetically engineered mouse models is to obtain the heterogeneity and the molecular and histopathologic characteristics of the parent primary tumors (10, 11). Moreover, the drug response characteristics of PDX are closely related to the patients’ clinical responses. PDX models have been reported in the treatment of many different types of solid tumors (12). It has been certified that PDX models can predict the patients’ chemotherapy response and provide guidance for informed clinical decision-making (13). So far, about 300 cases of 13 tumor types have been evaluated, and the overall agreement between the clinical and treatment response of PDX patients is 70 to 100% (14, 15). Although PDX has significant advantages, limitations prevent them from being widely used in personalized medicine. Tumor xenotransplantation takes too long, usually 4 to 8 months, and it takes extra time to generate enough tissue to test the treatment options in mice (16). Additionally, in many cancer types, the implantation rate in mouse models is usually less than 50%, and even lower in breast, prostate, and renal cell carcinoma (17). As a result, many patients with rapidly developing diseases are unable to benefit from PDX studies, and a fast and reliable alternative drug sensitivity assessment method is particularly urgent (18).

A rapid and accurate *in vivo* drug response detection method has been developed using hollow fiber implantation technology, which can effectively and realistically predict patients’ clinical responses to targeted therapy and chemotherapy. MiniPDX analysis provides a rapid and effective alternative to the PDX model for evaluating cancer treatment response that mimics the patients’ clinical treatment response. The simplified conditions in MiniPDX analysis enable tumor cells, especially primary tumor cells of various cancer types, to survive and grow in the body, thus achieving a high success rate (19–21). A PDX model establishment is a prerequisite for *in vivo* PDX analysis, usually takes several months, with the success rate usually much lower than 50%. However, MiniPDX analysis does not require establishing a PDX model in advance. This study will adopt the MiniPDX model from patients with gastric cancer with liver metastases (GCLM), screening sensitive drugs for patients with liver metastases from gastric cancer.

## Patients and Methods

### Patients Eligibility

Patients who were histologically confirmed with GCLM in Nanjing First Hospital and Qilu Hospital of Shandong University from January 2018 to June 2019 were enrolled consecutively in this cohort analysis. The criteria were as follow (1): 18 years of age or older (2); unresectable lesions with the necessity of systematic treatment (3); HER2 were negative (4); relapse or refractory to prior line treatment (4); Child-Pugh class A-B (5); ECOG PS of 0–2 (6); adequate organ function (white blood cell ≥3.9×10^9^/L, absolute neutrophil count ≥1.5×10^9^/L, platelets ≥100×10^9^/L, bilirubin ≤2 mg/dl; hemoglobin ≥10g/dl, and serum creatinine ≤150 mmol/L) (7); life expectancy of ≥3 months; and (6) received at least one response evaluation by CT or US. The exclusion criteria were as follows (1): patients who are indicated for liver resection (2), women with pregnancy or lactation (3), patients with a previous cerebrovascular event and active infectious disease (4), patients with clinically significant liver failure (i.e., encephalopathy or ascites found clinically).

This study was approved by the ethics committee of Nanjing First Hospital (KY20180604-05-KS-01). This research was conducted following the Declaration of Helsinki. All patients signed an informed consent.

### OncoVee™-MiniPDX Model

The chemotherapy regimens for patients in the MiniPDX group were based on drug sensitivity assay results in mice. The MiniPDX assay was performed using the OncoVee™-MiniPDX kit (LIDE Biotech Co., Ltd, Shanghai, China). Briefly, the tumor cell suspension from patients’ tumor tissues or biopsy samples was transferred to HBSS-washed capsules made of a hollow fiber membrane with an aperture of less than 500 kDa. The fiber system delivered the media to cells in a manner similar to blood delivery through the capillary network *in vivo*.

BALB/c nude mice (4–6 weeks of age) (SLARC Inc., Shanghai, China) weighing 15–20 g were selected for subcutaneous implantation. A small skin incision was made, and the OncoVee™-MiniPDX capsules were embedded in the subcutaneous tissues. One day after inoculation of tumor cells, the tumor-bearing mice were given the following drugs for 7 days [eg. gemcitabine, 60 mg/kg, ip, every 4 days; docetaxel, 10 mg/kg, ip, every 4 days; nab-paclitaxel, 20 mg/kg, intravenously (iv), every 4 days]. Normal saline was used as a control. Tumor cell viability was assessed based on relative fluorescence units (RFU) using CellTiter-Glo^®^ Luminescent Cell Viability Assay (Promega, Madison, WI, USA) to demonstrate the antitumor activity of each drug. The equation for calculating proliferation rate was as follows:


$$\text{Relative proliferation rate }(\text{T/C ratio})=\frac{(\text{RFUD}7-\text{RFUD}0)\text{drug}}{(\text{RFUD}7-\text{RFUD}0)\text{placebo}}$$

**Figure 1:**
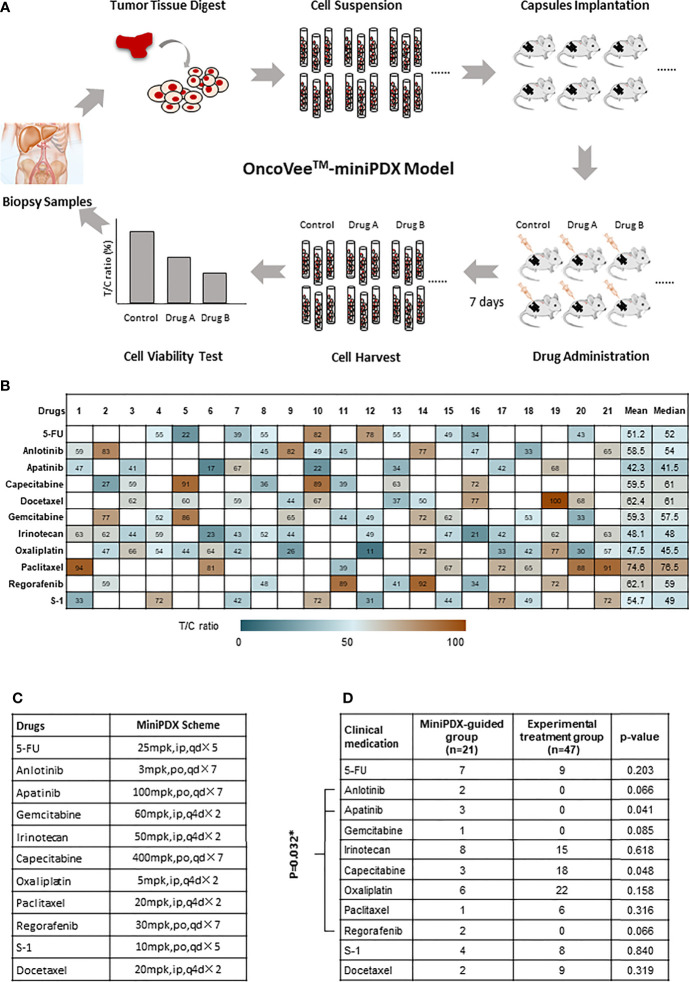
OncoVee™-MiniPDX flow diagram and the medication administration. **(A)** OncoVee™-MiniPDX flow diagram was as follows: Tumor cells, digested from biopsy samples (sometimes from fresh tissue), were loaded into three capsules and subcutaneously implanted in 4-week-old BALB/c nude mice. Then, 7 days after drug or placebo administration intraperitoneally or orally, capsules were harvested to evaluate drug sensitivity *via* cell viability test. According to the results of MiniPDX, the optimal regimens were selected for personalized chemotherapy. **(B)** Medication compliance and medication frequency were different from the control group. **(C)** Specific dosages, administration routes and cycles of drugs recommended by MiniPDX. * represents the multiplication sign, indicating the total number of days. **(D)** Assessment of the medication regimens of these two groups to evaluate the influence of MiniPDX on drug selection in clinical practice. * represents statistical significance, p < 0.05.

T/C ratio was defined as the relative proliferation rate of the treatment group compared with the control group 7 days after drug administration. A T/C ratio less than 50% was considered as the cutoff value to indicate response, which was proven before (22). The research flow chart is shown in **Figure 1**. All procedures were performed in accordance with the guidelines for the Care and Use of Laboratory Animals of the National Institutes of Health in the absence of specific pathogens.

### Conventional Chemotherapy

Patients in the conventional group were treated with chemotherapy regimens according to National Comprehensive Cancer Network (NCCN) Clinical Practice Guidelines in Oncology, version 1.2018. Treatment regimens were decided by at least two independent medical professionals.

### Outcomes and Measurement

The primary endpoint was the overall survival (OS) of included patients. The secondary endpoints included progression-free survival (PFS), objective response rate (ORR), disease control rate (DCR), and biomarkers response status. During the treatment, patients were followed up every month, then every 3 months after treatment till death or loss. The follow-up evaluations consisted of history, physical examination, hematology and blood chemistry panels, including serum tumor markers. Progression-free survival (PFS) and OS were measured as the time between treatment initiation and documented disease progression (PFS) or death (OS). OS refers to the time from treatment initiation to death. PFS is the time from treatment initiation to disease progression or death. All patients underwent conventional CT scans of liver by Somatom PLUS-S CT scanner (Siemens Medical Systems, Erlangen, Germany) at baseline and during follow-up. CT images were processed using 3D slice software package (Version 4.7). At least two radiologists with more than 10 years of work experience and an assistant researcher completed the entire process together. Radiographic assessments of short-term efficacy were performed every two cycles until disease progression or death during chemotherapy as per RECIST v1.1, and patients were classified into four subgroups: complete remission (CR), partial remission (PR), stable disease (SD), and progressive disease (PD). ORR was defined as the percent of patients with CR and PR from all the patients. And DCR was defined as the percent of the patients who achieved CR, PR, and SD.

### Statistical Analysis

All the data analyses and plots were conducted using the statistical software of STATA Version 13.0 (College Station, TX, USA). Our data were described as the mean ± SD for normally distributed data or median with range for non-normally distributed data. Continuous variables with normally distributed were analyzed using unpaired Student’s t-test. For multiple comparisons, the Tukey-Kramer honestly significant difference test was applied following ANOVA. OS analysis of patients was conducted by the Kaplan-Meier method. Potential independent risk factors for survival were evaluated by univariate analysis (log-rank test) and multivariate analysis (Cox proportional hazards model). P-value < 0.05 indicated statistical significance. The OS and PFS were analyzed using the Kaplan-Meier method and log-rank test. The correlations between clinical-pathological variables and drug sensitivity were analyzed using the Pearson χ2 test. P<0.05 was considered to indicate a statistically significant difference.

## Results

### Baseline Characteristics of Patients

According to the inclusion criteria, 21 patients who received OncoVee™-MiniPDX drug sensitivity test were included. As a control group, 47 cases who received experimental treatment according to the NCCN guidance without the results of the MiniPDX model were concurrently selected. As present in **Table 1**, the baseline characteristics of these two cohorts were balanced without statistical difference. The previous line treatments include SP (S-1 and cisplatin), CP (irinotecan and cisplatin), DCF (Docetaxel and cisplatin and 5-FU), FP (5-FU and cisplatin), FOLFIRI (5-FU and leucovorin and irinotecan), XELOX (oxaliplatin and capecitabine), in which SP or CP was mainly used (60%). Relapse disease count for 38% of all these patients, with a median time to relapse of 3.2 (range:0.5–5.8) months.

**Table 1:** Patients’ demography and tumor characteristics.

Characteristics	MiniPDX-guided group (n = 21)	Experimental treatment group (n = 47)	p-value
Age, years			0.270
Median (range)	62 (28–83)	63 (32–86)	
<65, n (%)	8	20	
≥65	13	27	
Sex			0.612
Male	13	26	
Female	8	21	
ECOG PS			0.560
0–1	17	35	
2	4	12	
Primary gastric tumors size			0.763
Mean (SE), cm	5.76 (2.35)	5.49 (2.96)	
<5 cm	9	22	
≥5 cm	12	25	
Differentiation of primary tumor			0.954
Well	3	8	
Moderate	15	32	
Poor	3	7	
T-stage of primary tumor^&^			0.934
pT1	2	7	
pT2	5	11	
pT3	11	22	
pT 4	3	7	
N-stage of Primary tumor^&^			0.908
N0	3	7	
N1	9	19	
N2	6	11	
N3	3	10	
Number of metastases			0.634
Median (range)	4 (1–9)	4 (1–11)	
Solitary n (%)	9	22	
2–5, n (%)	9	15	
>5, n (%)	3	10	
Metastases tumors size			0.793
Median (range), cm	4.77 (2.18)	4.06 (2.69)	
<5 cm	10	24	
≥5 cm	11	23	
Metastases lesions location			0.914
Left lobe, n (%)	5	10	
Right lobe, n (%)	6	12	
Both, n (%)	10	25	
Interruption of hepatic hilum			0.243
Yes	5	18	
No	16	29	
Relapse or refractory disease			0.600
Relapse	9	17	
Refractory	12	30	
CEA level*			0.210
Mean ± SE, ng/ml	47.66 ± 29.06	54.01 ± 33.72	
Negative	4	16	
Positive	17	31	
CA199^#^			0.349
Mean ± SE, U/ml	3879.2 ± 1823.3	4211.3 ± 2201.3	
Negative	6	19	
Positive	15	28	

^&^Tumor stage was defined according to the American Joint Committee on Cancer (AJCC) TNM staging system (AJCC 7^th^ edition).

*CEA levels were measured in 16 and 38 patients, respectively, in MiniPDX-guided and experimental treatment groups. A CEA level of <5 ng/ml was considered as negative.

^#^CA19-9 levels were measured in 17 and 40 patients, respectively, in MiniPDX-guided and experimental treatment groups. A CA19-9 level of <37 U/ml was considered as negative.

CA19-9, carbohydrate antigen 19-9; CEA, carcinoma embryonic antigen; ECOG PS, Eastern Cooperative Oncology Group physical status; PDX, patient-derived xenograft.

### Efficacy Prediction and Medication Regimens by MiniPDX Model

As presented in **Figure 1B**, the sensitivity of 11 kinds of drugs, including 5-FU, Anlotinib, Apatinib, Capecitabine, Docetaxel, Gemcitabine, Irinotecan, Oxaliplatin, Paclitaxel, Regorafenib, and S-1, was tested in patients in the MiniPDX-guided group. Based on the results of MiniPDX, Apalitinib, Irinotecan, and Oxaliplatin seemed to show potential efficacy in the susceptibility tests, with both mean and median pooled T/C ratio less than 50%.

When patients were taken as subjects for analysis, 17 out of 21 patients were clinically administrated according to the results of MiniPDX tests, in whom at least one kind of drug with T/C less than 50%, which were considered the potential efficacy drugs (except for case #4, case #14, case #19, and case #21) (**Figures 1B, C**). The medication compliance to MiniPDX from physicians or patients was estimated to be 80.95%.

In addition, the medication regimens of these two groups were also assessed to evaluate the influence of MiniPDX on drug selection in clinical practice. With the exception of capecitabine (marginal difference P =0.048), the results showed no statistical difference between the MiniPDX group and patients receiving experimental treatment (**Figure 1D**). However, seven patients in the MiniPDX group received targeted drugs, including Anlotinib, Apatinib, and Regofenib, compared with no administration in the control group. Chi test showed significant difference (33.3 *vs.* 0%, P =0.032). The increased use of targeted drugs might contribute to the survival benefit.

### Survival Outcomes and Subgroup Analysis

**Figure 2:**
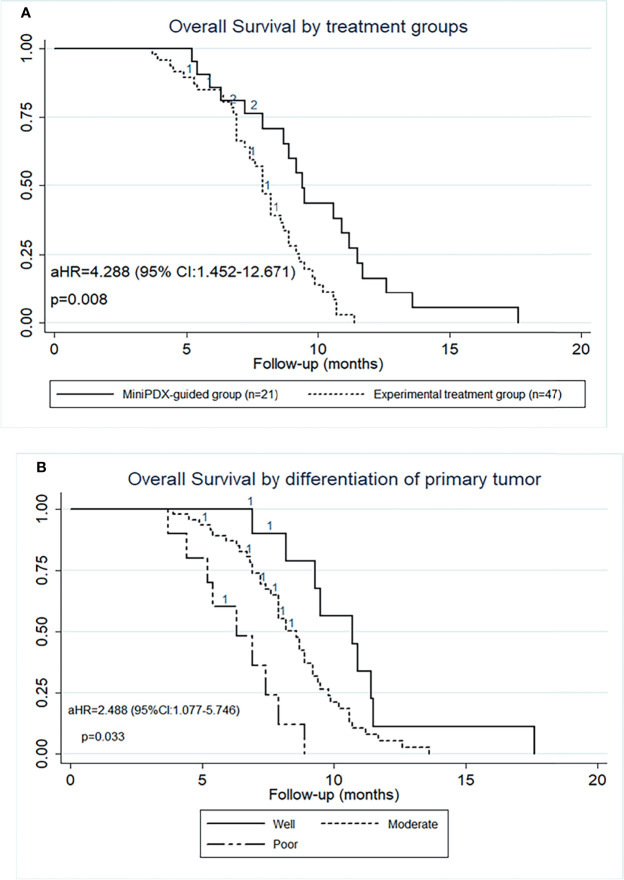
OS outcomes of patients included in this study. **(A)** OS was significantly prolonged in cases who received the MiniPDX-guided regimens compared with the control group. **(B)** The poor-differentiation primary tumor was an independent risk factor for poor prognosis.

The median OS of the MiniPDX-guided group was estimated to be 9.4 months with 95% confidence interval (CI) of 7.9–11.2 months. Meanwhile, patients with experimental treatment had a median OS of 7.9 (95% CI: 7.2–8.7) months (**Figure 2A**). Log-rank test revealed a statistical difference between these two groups (HR=2.586, 95% CI=1.362–4.908, p=0.004) (**Table 2**). The 6- and 12-month survival rates were 78.9, 36.9, and 55.7, 17.8%, respectively, in the minPDX group and control group.

**Table 2:** Univariate and multivariate Cox proportional hazards regression analysis of overall survival.

Characteristics	Median OS months	Univariate HR (95% CI)	P-value	Multivariate aHR^&^(95% CI)	P-value^&^
Treatment		2.586 (1.362–4.908)	0.004	4.288 (1.452–12.671)	0.008
MiniPDX-guided group, n=21	9.4 (7.9–11.2)				
Experimental treatment group, n=47	7.9 (7.2–8.7)				
Age		1.613 (0.947–2.747)	0.078	NA	NA
<65, n=31	8.9 (7.9–9.5)				
≥65, n=37	7.4 (6.9–8.7)				
Sex		0.747 (0.442–1.263)	0.277	NA	NA
Male, n=39	8.2 (6.9–9.2)				
Female, n=29	8.6 (7.9–9.9)				
ECOG PS		1.294 (0.710–2.359)	0.400	NA	NA
0–1, n=52	8.7 (7.4–9.2)				
2, n=16	8.2 (7.2–9.5)				
Primary gastric tumors size		2.008 (1.163–3.469)	0.012	1.624 (0.674–3.915)	0.280
<5 cm, n=31	8.9 (8.2–10.2)				
≥5 cm, n=37	7.6 (6.9–8.9)				
Differentiation of primary tumor		2.780 (1.612–4.791)	0.000	2.488 (1.077–5.746)	0.033
Well, n=11	10.7 (6.9–11.5)				
Moderate, n=47	8.6 (7.6–9.2)				
Poor, n=10	6.3 (3.7–7.9)				
T-stage of primary tumor		1.211 (0.887–1.654)	0.277	NA	NA
pT1, n=9	8.9 (7.2–10.6)				
pT2, n=16	8.9 (6.9–10.7)				
pT3, n=33	8.2 (7.4–9.2)				
pT4, n=10	6.9 (5.3–8.7)				
N-stage of primary tumor		1.441 (1.087–1.912)	0.011	1.467 (1.007–2.138)	0.046
N0, n=10	9.3 (6.9–10.9)				
N1, n=28	8.2 (7.4–9.4)				
N2, n=17	7.9 (6.3–9.5)				
N3, n=13	7.6 (4.9–8.7)				
Number of metastases		1.149 (0.820–1.639)	0.426	NA	NA
Solitary, n=31	9.8 (6.3–11.2)				
2–5, n=24	9.2 (6.8–9.9)				
>5, n=13	8.2 (7.4–8.9)				
Metastases tumors size		1.830 (1.078–3.107)	0.025	1.351 (0.589–3.103)	0.478
<5 cm, n=34	9.2 (7.9–9.9)				
≥ 5cm, n=34	7.6 (6.4–8.7)				
Metastases lesions location		0.876 (0.627–1.224)	0.438	NA	NA
Left lobe, n=15	7.9 (4.9–9.8)				
Right lobe, n=18	8.2 (7.9–8.9)				
Both, n=35	8.6 (7.2–9.5)				
Interruption of hepatic hilum		0.599 (0.335–1.071)	0.084	NA	NA
Yes, n=24	7.9 (6.8–8.2)				
No, n=44	8.9 (7.9–9.4)				
Relapse or refractory disease		1.136 (0.736–1.563)	0.535	NA	NA
Relapse, n=26	8.6 (6.8–10.7)				
Refractory, n=42	8.2 (7.4–9.9)				
CEA level*		0.810 (0.461–1.425)	0.466	NA	NA
Negative, n=16	8.6 (6.9–9.2)				
Positive, n=38	8.2 (7.4–9.4)				
CA199^#^		1.021 (0.585–1.781)	0.942	NA	NA
Negative, n=17	8.6 (7.6–9.3)				
Positive, n=40	7.9 (7.2–9.4)				

*CEA levels were measured in 16 and 38 patients, respectively, in MiniPDX-guided and experimental treatment groups. A CEA level of <5 ng/ml was considered as negative.

^#^CA19-9 levels were measured in 17 and 40 patients, respectively, in MiniPDX-guided and experimental treatment groups. A CA19-9 level of <37 U/ml was considered as negative.

^&^These results were adjusted by multiple variables identified in univariate analyses, including treatment group, primary gastric tumors size, differentiation of primary tumor, N-stage of primary tumor, metastases tumors size.

CA19-9, carbohydrate antigen 19-9; CEA, carcinoma embryonic antigen; ECOG PS, Eastern Cooperative Oncology Group physical status; PDX, patient-derived xenograft; OS, overall survival; NA, not applicated.

To explore the survival outcomes in more detail, subgroup analyses based on the baseline characteristics were conducted (**Table 2**). The univariate analysis revealed that patients who received MiniPDX-guided treatment, with primary tumor size less than 5 cm, with well- or moderated-differentiation tumor, and with hepatic metastases less than 5 cm were associated with improved survival outcomes of patients. Multivariate regression analyses suggested that treatment without MiniPDX test, poor-differentiation of the primary tumor (**Figure 2B**), N3 stage of the primary tumor were independent risk factors for the poor prognosis.

### RECIST Response Status and Biomarkers Response

**Figure 3:**
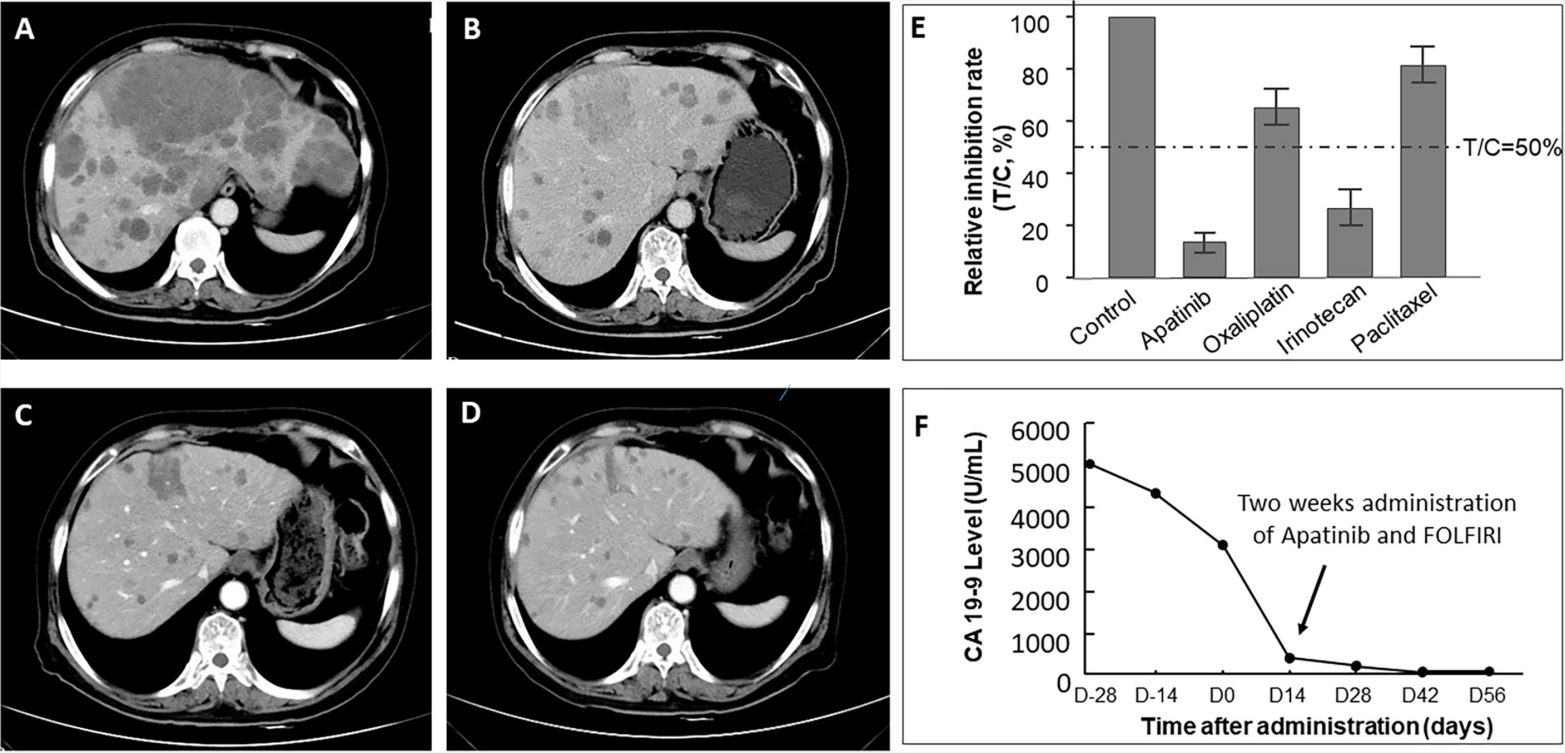
CT images of one 67-year-old patient who received MiniPDX-guided regimen. **(A)** The patient suffered a progressive disease after six cycles of XELOX as first-line treatment. **(E)** The MiniPDX test revealed that this patient was resistant to Paclitaxel and Oxaliplatin and was sensitive to Irinotecan and Apatinib. **(B)** Two cycles after second-line Apatinib and FOLFIRI (Irinotecan and 5-FU) treatment, hepatic metastases shrank obviously. **(C)** Three cycles and **(D)** four cycles after Apatinib and FOLFIRI administration, lesions in the liver continue to shrink. **(F)** CA19-9 levels significantly decrease to negative (<37 U/ml) after 2 weeks of Apatinib and FOLFIRI administration and maintain low levels in the follow-up period.

All the patients in these two groups received at least one RECIST evaluation after systematic treatment. Seventeen out of all 21 patients in the MiniPDX group was indicated at least one kind of potential drug use based on drug sensitivity tests. The correlation of the response rate between the MiniPDX test and the clinical response status was 70.6% (11/17) in these patients. ORR of the MiniPDX-guided group was 57.14%, which was significantly higher than 25.53% in the control group (p=0.029). Similarly, the DCR was also considerably improved in the MiniPDX group (85.71 *vs.* 68.08%, p=0.035). In addition, more patients experienced a CEA response in the MiniPDX-guided group (62.50 *vs.* 37.84%). However, the difference did not reach statistical significance (p=0.174). Meanwhile, treatment with MiniPDX-guided drugs was associated with improved CA19-9 response status compared with the control group (p=0.009) (**Table 3**). **Figure 3** shows the CT images and the CA19-9 response status of one 67-year-old patient who received MiniPDX-guided regimen.

**Table 3:** Metrics of RECIST response, CEA response, and CA 19-9 response to different groups.

Characteristics	MiniPDX-guided group (n = 21)	Experimental treatment group (n = 47)	p-value
**RECIST 1.1**			0.038
CR	0	0	
PR	12 (57.14%)	12 (25.53%)	
SD	6 (28.57%)	20 (42.55%)	
PD	3 (14.29%)	15 (31.91%)	
ORR	12 (57.14%)	12 (25.53%)	0.029
DCR	18 (85.71%)	32 (68.08%)	0.035
CEA parameters*			0.174
Decrease >50%	10 (62.50%)	14 (37.84%)	
Decrease >20%	4 (25.00%)	12 (32.43%)	
Decrease <20% or increase	2 (12.50%)	11 (29.73%)	
**CA 19-9 parameters^#^**			0.009
Decrease >50%	12 (70.59%)	12 (30.0%)	
Decrease >20%	2 (11.76%)	18 (45.00%)	
Decrease <20% or increase	3 (17.65%)	10 (25.00%)	

*CEA levels were evaluated in 16 and 37 patients, respectively, in the MiniPDX-guided and control groups.

^#^CA19-9 levels were tested in 17 and 40 patients, respectively, in the MiniPDX-guided and control groups.

CA19-9, carbohydrate antigen 19-9; CEA, carcinoma embryonic antigen; CR, complete response; DCR, disease control rate; ECOG PS, Eastern Cooperative Oncology Group physical status; ORR, objective response rate; PD, progressive disease; PDX, patient-derived xenograft; PR, partial response; SD, stable disease.

## Discussion

Most gastric cancer patients with concomitant liver metastases were excluded from being candidates for curative surgery accompanied by hepatic resection due to the simultaneous presence of incurable factors such as peritoneal dissemination, widespread lymph nodal metastasis, and direct invasion to adjacent structures (23). In fact, hepatic metastases to gastric cancer usually represent only a fraction of the broader spread of the primary tumor. In our research, tumor cells were enriched from biopsy samples of 21 patients with GCLM, followed by establishing a MiniPDX model and the formulation of individualized chemotherapy regimens based on drug sensitivity test results. The results confirmed that MiniPDX-guided chemotherapy was more beneficial to GCLM patients than conventional treatment, which might have some implications for oncologists making informed decisions about individualized chemotherapy.

For patients who relapsed or were refractory to first-line treatment (e.g., 5-FU and platinum), second-line chemotherapy regimes, including SPA (S-1 and Paclitaxel) (24), XELOX (capecitabine and oxaliplatin) (25, 26), DOCOX (Docetaxel plus oxaliplatin) (27), S-1 monotherapy, XELIRI (capecitabine and irinotecan) (28), and some newly developed targeted drugs (e.g., Apatinib monotherapy) (29), have failed to show an adequate response to them. The OS and PFS were pooled to be approximately 7.0 and 4.5 months, respectively, in advanced gastric cancer. Our results on the control group showed a median OS and PFS of 7.9 and 4.2 months, which is in accordance with previous results, and indirectly confirmed the robust results of this study.

PDX models, either heterotopic or orthotopic implantation, allow invaluable assessment of human tumor biology, therapeutic targets, and drug evaluation based on the principle of biological stability and accurately reflecting the tumor characteristic of patients (30–32). But lengthy test period and unsatisfactory engraftment rate prevent the wide application of PDX in some high-grade malignant tumors, especially in gastric cancer (33). MiniPDX is a rapid, systematic *in vivo* assay to measure drug sensitivity of tumor cells and takes only 7 days. As Zhang et al. reported, MiniPDX could overcome the limitations of PDX and retain the accuracy and efficiency, compared to PDX models, with 92% of positive value, 81% negative value, 80% sensitivity, and 90% specificity (19).

The clinical application of MiniPDX has become more prevalent in recent years; increasing encouraging results on MiniPDX were reported (34). Zhan et al. used MiniPDX to guide the selection of chemotherapeutic regimens in patients with gallbladder carcinoma, who had significantly longer median PFS (17.6 months *vs.* 12.0 months, P=0.014) and overall survival (18.6 months *vs.* 13.9 months, P=0.030) than patients with conventional chemotherapy (20). In another case reported by Zhao et al., personalized treatment based on MiniPDX and whole-exome sequencing in a patient with metastatic duodenal adenocarcinoma demonstrated that this combination could rapidly assess drug sensitivity and reveal significant genetic alterations (21). Also, the study by Yang et al. showed a significant benefit from the MiniPDX test than the control group in hepatocellular carcinoma (DFS: 25.8 months *vs.* 18.2 months, P=0.022) (35). Similar results were validated in ovarian cancer (36) and lung cancer (37). In our study, individual chemotherapy based on MiniPDX also showed superiority to prolong the OS and PFS of patients with GCLM, which could consider solid validation evidence for previous studies.

In terms of the response status, our study showed that ORR and DCR were also higher in the MiniPDX group than in the experimental treatment group. Moreover, the biomarkers’ levels of CEA and CA19-9 have also achieved a better response status in the MiniPDX group. The correlation of the response rate between the MiniPDX test and the clinical response status was estimated to be 70.6% in those MiniPDX, indicating a T/C ratio of less than 50%. Considering the other four cases, two patients did not achieve a clinical response with drugs in the test list.

It should be noted that the MiniPDX test significantly increased the selection of targeted drugs, including Apatinib, Anlotinib, and Regorafenib. It might contribute to the response and survival benefit of patients who received MiniPDX-guided therapy. This suggested that MiniPDX is not about finding the more potent drugs, but about finding the more appropriate drugs for individuals. This concept is to fully respect the tumor heterogeneity of the patients to achieve personalized treatment. The difference between these two groups was not significant to each drug individually, which may be due to the limited sample size and statistical power. Moreover, based on the baseline tumor characteristics in the present study, we found that our enrolled patients had predominantly moderately to poorly differentiated tumors in both observasion and control groups (18/21 *vs.* 40/47), which indicates a poor prognosis in clinical practices. Subsequent multivariate analysis also showed that moderately to poorly differentiated tumor was an independent risk factor for poor prognosis after adjustment by the MiniPDX application (P =0.033). The above results suggested that MiniPDX, although showing statistically promising results for the overall cohort, did not overcome the inherent independent risk factors, like moderately or poorly differentiated tumors, similarly, N-stage of N1 to N3.

Some limitations of this research should be acknowledged. Firstly, the limited number of participants, especially in the MiniPDX group, may affect the reliability and statistical power of this analysis. Secondly, the timing of MiniPDX testing, whether it should be performed in first-line or second-line therapy, needs further discussion. Therefore, the conclusions of this study need to be further verified in a randomized controlled clinical trial with a larger sample size. Nevertheless, our research indicated the MiniPDX-guided chemotherapy regimen selected the most effective drugs or regimens to treat GCLM patients and could effectively improve patient outcomes. Our results might provide a meaningful and exploratory basis for the precise treatment of GCLM and even other solid tumors in the future.

In conclusion, treatment based on MiniPDX is promising to improve the survival and response of GCLM patients in this preliminary study. OncoVee™-MiniPDX models have potential in the treatment of other aggressive tumors. However, further well-designed clinical trials with a larger sample size are necessary to verify the results of this study.

## Data Availability Statement

The raw data supporting the conclusions of this article will be made available by the authors, without undue reservation.

## Ethics Statement

The studies involving human participants were reviewed and approved by the ethics committee of Nanjing First Hospital. The patients/participants provided their written informed consent to participate in this study. The animal study was reviewed and approved by the ethics committee of Nanjing First Hospital. Written informed consent was obtained from the individual(s) for the publication of any potentially identifiable images or data included in this article.

## Author Contributions

All authors contributed to the conception and design. XW and JS supervised the study. Material preparation, data collection, and analysis were performed by YG and XZ. YG, XZ, WL, CT, and DG drafted the manuscript. XW and JS critically revised the manuscript for important intellectual content. All our authors contributed to the article and approved the submitted version.

## Funding

This study was supported by the National Natural Science Foundation of China (No. 81773240), the Nanjing Medical Science and Technique Development Foundation (No. QRX17062), and the Nanjing Health Science and Technology Development Foundation (No. JQX18004).

## Conflict of Interest

The authors declare that the research was conducted in the absence of any commercial or financial relationships that could be construed as a potential conflict of interest.

## Publisher’s Note

All claims expressed in this article are solely those of the authors and do not necessarily represent those of their affiliated organizations, or those of the publisher, the editors and the reviewers. Any product that may be evaluated in this article, or claim that may be made by its manufacturer, is not guaranteed or endorsed by the publisher.
